# Characterization of cell line with dedifferentiated GIST‐like features established from cecal GIST of familial GIST model mice

**DOI:** 10.1111/pin.13315

**Published:** 2023-02-24

**Authors:** Daisuke Sano, Takako Kihara, Jiayin Yuan, Neinei Kimura, Mizuka Ohkouchi, Yuka Hashikura, Shuichi Ohkubo, Seiichi Hirota

**Affiliations:** ^1^ Department of Surgical Pathology Hyogo Medical University School of Medicine Nishinomiya Japan; ^2^ Department of Pathology The First People's Hospital of Foshan Foshan Republic of China; ^3^ Discovery and Preclinical Research Division Taiho Pharmaceutical Co. Ltd Tsukuba Japan

**Keywords:** cell line establishment, dedifferentiated GIST, familial GIST model mouse, Pimitespib, Telaglenastat

## Abstract

Approximately 40 families with multiple gastrointestinal stromal tumors (GISTs) and germline c‐*kit* gene mutations have been reported. Three knock‐in mouse models have been generated, and all the models showed a cecal GIST. In the present study, we established a cell line derived from cecal GIST in a familial GIST model mouse with KIT‐Asp818Tyr. Since the established cells showed spindle‐shaped morphology with atypical nuclei, and since immunohistochemistry revealed that they were positive for α‐SMA but negative for KIT, CD34 and desmin, the phenotypes of the cells were reminiscent of dedifferentiated GIST‐like ones but not the usual GIST‐like ones. Gene expression analysis showed that the cell line, designated as DeGISTL1 cell, did not express c‐*kit* gene apparently, but highly expressed *HSP90* families and *glutaminase 1*. Pathway analysis of the cells revealed that metabolic pathway might promote their survival and growth. Pimitespib, a heat shock protein 90α/β inhibitor, and Telaglenastat, a selective glutaminase 1 inhibitor, inhibited proliferation of DeGISTL1 cells and the combination of these showed an additive effect. DeGISTL1 cells might be a good model of dedifferentiated GISTs, and combination of Pimitespib and Telaglenastat could be a possible candidate for treatment strategy for them.

**Abbreviations**
GISTs: gastrointestinal stromal tumors
GLS: glutaminase
HSP: heat shock protein
IM: imatinib mesylate
KEGG: Kyoto Encyclopedia of Genes and Genomes
PDGFRA: platelet derived growth factor receptor alpha
TK: tyrosine kinase

## INTRODUCTION

Gastrointestinal stromal tumors (GISTs) are the most common mesenchymal tumors of the human gut. Most GISTs express a receptor tyrosine kinase (TK) known as KIT, and most of the sporadic GISTs have a somatic c‐*kit* gene mutation which is considered to be a cause of GIST.^1^ The somatic mutations are frequently present at exon 11, but they are also found at exon 8, 9, 13, or 17. Approximately 40 families with multiple GISTs and a germline mutation of the c‐*kit* gene have been described,^2^, ^3^ and the c‐*kit* gene mutations are similarly found at exon 8, 11, 13, or 17. Three knock‐in mouse models of the familial GISTs with mutation at exon 11, 13, or 17 have been generated, and among them the mouse model with exon 17 KIT‐Asp818Tyr corresponding to human KIT‐Asp820Tyr was generated by us.^4^ In all types of the mouse models, a cecal GIST develops.

So far, cell lines with GIST characteristics derived from cecal GISTs of these three model mice of familial GISTs have not been established yet. We tried to establish a GIST cell line from cecal GIST of a model mouse with exon 17 KIT‐Asp818Tyr. Although we obtained a cell line from the primary culture of the cecal GIST, the established cells showed undifferentiated sarcoma‐like morphology. There is a possibility that the cells are derived from dedifferentiation of cecal GIST cells.^5^ We examined characteristics of the cells, and investigated the antitumor effects of Pimitespib,^6^ a heat shock protein 90 inhibitor, and Telaglenastat,^7^ a glutaminase (GLS) 1 inhibitor, as therapeutic candidates for dedifferentiated GIST‐like cells.

## MATERIALS AND METHODS

### Cell line establishment from familial GIST model mouse

Cecal GIST tissue was dissected from a homozygous model mouse of multiple GIST family with KIT‐Asp818Tyr. The tumor tissue was cut into 1 mm cubes, and primary cell culture was done in DMEM supplemented with GlutaMAX™ and 10% fetal bovine serum (FBS). Cells which came out of the cubic tissue were cultured and passaged.

### Cell proliferation assay

A cell line established from the cecal GIST tissue (DeGISTL1 cells) was plated in 24‐well plates (CORNING, Corning, NY, USA) at 5 × 10^3^ cells per well in growth medium. After incubation for 1, 2, 3, 4, and 5 days, cells were trypsinized, resuspended in Accumax (Innovative Cell Technologies) and counted by hemocytometer (Z1, Beckman Coulter). Six wells were used for each cell type in each experiment. Cell proliferation assay was repeated three times.

### RNA extraction, gene expression analysis and pathway analysis

Total RNA was extracted from DeGISTL1 cells using RNeasy Mini Kit (Qiagen, Hilden, Germany) according to the manufacturer's recommendation. Gene expression profiles of the cell line was examined using GeneChip expression analysis (Affymetrix). Pathway of the cell line was identified using by Kyoto Encyclopedia of Genes and Genomes (KEGG).

### Transplantation of DeGISTL1 cells to mice

Cultured DeGISTL1 cells (1 × 10^6^ cells) were transplanted into the abdominal cavity of five C57BL/6 wild‐type mice with same background of the model mice. After DeGISTL1 cells formed a tumor, the mice were killed by cervical dislocation which was performed by the technically skilled persons prior to tumor dissection. Animal experiment protocols were approved by the Hyogo Medical University Animal Experiment Committee (No. 20‐063), and all animal experiments were conducted according to the institutional ethical guidelines for animal experimentation in Hyogo Medical University.

### Histological analysis

Cell block of cultured DeGISTL1 cells made after stripping them off from culture dishes and dissected tissues after transplantation of DeGISTL1 cells to mouse peritoneum were fixed in 10% buffered formalin and embedded in paraffin. Three‐micrometer‐thick sections were cut and stained with hematoxylin and eosin (H&E). Immunohistochemistry (IHC) for mouse anti‐desmin monoclonal Ab (DE‐R‐II, Leica, 1:500), mouse anti‐α‐SMA monoclonal Ab (1A4, Dako Cytomation, 1:500), mouse anti‐CD34 monoclonal Ab (QBEnd/10, Leica, 1:2000), rabbit anti‐S‐100 protein polyclonal Ab (Leica, 1:5000) and rabbit anti‐KIT polyclonal Ab (A4502, DAKO Cytomation, 1:200) was performed using Bond Polymer Refine Detection (Leica).

### Inhibitory effect of glutamine deprivation on proliferation of DeGISTL1 cells

The DeGISTL1 cells (5 × 10^3^ cells/well) were cultured in glutamine‐free DMEM with 10% FBS to examine the glutamine dependency of the DeGISTL1 cells. The cell numbers were counted and viability was evaluated.

### Inhibitory effects of Telaglenastat, Imatinib and Pimitespib on proliferation of DeGISTL1 cells

Telaglenastat (CB‐839), a GLS1 inhibitor, purchased from MedChemExpress Co. Ltd. was added to culture media of DeGISTL1 cells (5 × 10^2^ cells/well) at various concentrations (0, 1, 2.5, 5, and 10 μM) for 5 days to examine the glutamine dependency of the DeGISTL1 cells. We also examined the effect of Telaglenastat on DeGISTL1 cells (5 × 10^3^ cells/well) at various concentrations (0, 5, and 10 μM) for 72 h. Imatinib (Novartis Pharma KK), an inhibitor of TKs including KIT, was added to culture media of DeGISTL1 cells (5 × 10^3^ cells/well) at various concentrations (0, 0.25, 0.5, 1, and 5 μM) to examine the effect of TK inhibition on DeGISTL1 cells at 5 day. Pimitespib (Taiho Pharmaceutical Co. Ltd.), also called TAS‐116, which is a HSP 90 inhibitor, was added to culture media of DeGISTL1 cells (5 × 10^3^ cells/well) at various concentrations (0, 0.25, 0.5, 1, and 5 μM) after dissolving in stearoxy hydroxypropyl methylcellulose (Daido Chemical Co. Ltd.) to examine its effect on DeGISTL1 cells showing high expression of HSP90 families at 5 day. Effect of combination of Pimitespib (0.25 μM) and Telaglenastat (0.15 μM) on DeGISTL1 cells (5 × 10^3^ cells/well) was also examined at 5 day. In these experiments, culture media used was DMEM supplemented with GlutaMAX™ and 10% FBS.

### Statistical analysis

For comparison of cell proliferation between agent‐treated DeGISTL1 cells and nontreated DeGISTL1 cells, the mean and standard deviation (SD) were calculated for each group and the significance of differences (*p* < 0.05) between groups was determined by the Student's *t*‐test using GraphPad Prism 9.4.1 software.

## RESULTS

### Cell line establishment from familial GIST model mice

**Figure 1:**
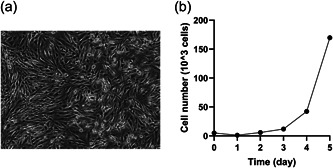
Morphology of the established cell line and its proliferation curve. (a) Morphology of the established cell line was shown under phase‐contrast microscopy. (b) The established cultured cells (5 × 10^3^ cells) were seeded each four well in a 24‐well plate, and counted for up to 5 days. The doubling time calculated from growth curve was 12.5 h.

Cells obtained from primary culture of the cecal GIST tissue of the homozygous knock‐in mouse was passaged. Passage was repeated over 30 times. After 30 repeats of the passage, a cell line was considered to be established (Figure 1a). We confirmed that the cells have homozygous KIT‐Asp818Tyr mutation in genome level (Supporting Information: Supplementary Figure 1), indicating that they are definitely from the homozygote model mouse tissue. The doubling time (12.5 h) was calculated using the proliferation curve (Figure 1b).

### GeneChip expression analysis of DeGISTL1 cells

**Figure 2:**
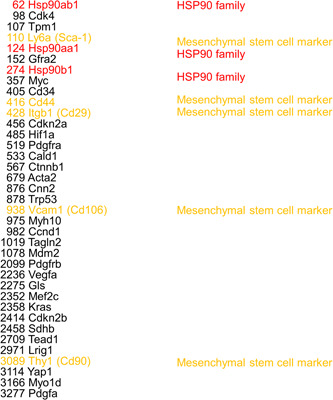
GeneChip expression analysis of the established cell line (list of highly expressed genes). The cultured cells had high‐level gene expression of *Hsp90ab1*, *Hsp90aa1*, and *Hsp90b1* (red), and murine mesenchymal stem cell marker genes (orange). *Acta2*, encoding alpha‐smooth muscle actin (α‐SMA), and *Gls*, encoding glutaminase 1, were also shown to be abundantly expressed by them.

The RNA extracted from the established cells was used for gene expression analysis. They showed high‐level expression of *Hsp90ab1*, *Hsp90aa1*, and *Hsp90b1* genes (Figure 2). They also highly expressed murine mesenchymal stem cell marker genes, such as *Ly6a (Sca‐1)*, *Cd44*, *Itgb1 (Cd29)*, *Vcam1 (Cd106)*, and *Thy1 (Cd90)* (Figure 2). *Acta2*, encoding alpha‐smooth muscle actin (α‐SMA), and *Gls*, encoding glutaminase 1, were also shown to be abundantly expressed by them (Figure 2). Very low expression of c‐*kit* and *Dog1* was observed (data not shown).

### KEGG pathway analysis of DeGISTL1 cells

**Figure 3:**
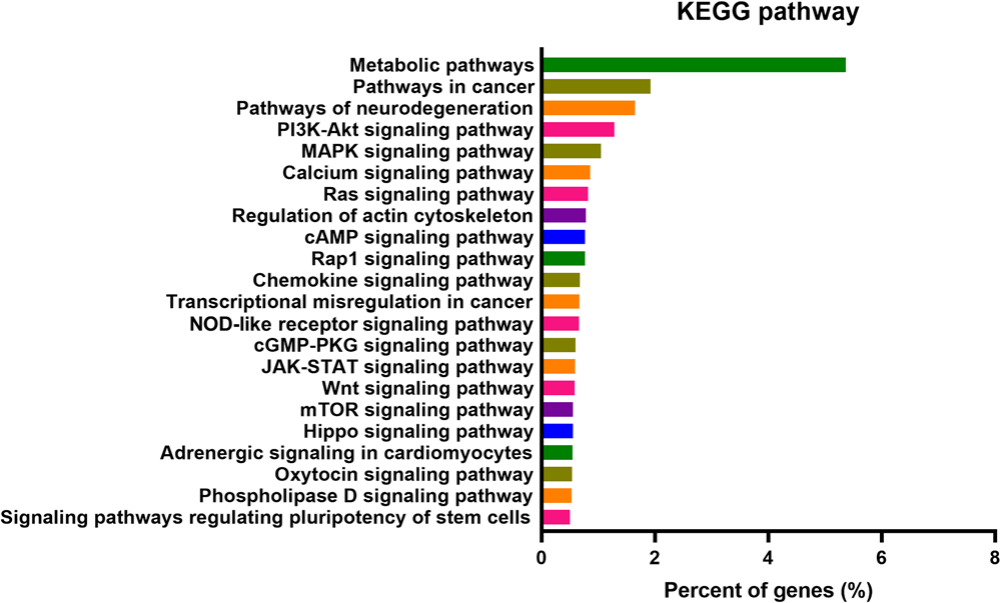
KEGG pathway analysis of the established cell line. The cells showed high expression of metabolic pathway genes and pathway genes in cancer by KEGG pathway analysis. Metabolic pathway is considered to promote survival and growth of them.

The established cells showed high expression of metabolic pathway genes and pathway genes in cancer by KEGG pathway analysis. Prominent gene expression in metabolic pathway including *Gls* is considered to promote survival and growth of them (Figure 3).

### Histological examinations of cell block of established cultured cells and tumor formed after transplantation to mice

**Figure 4:**
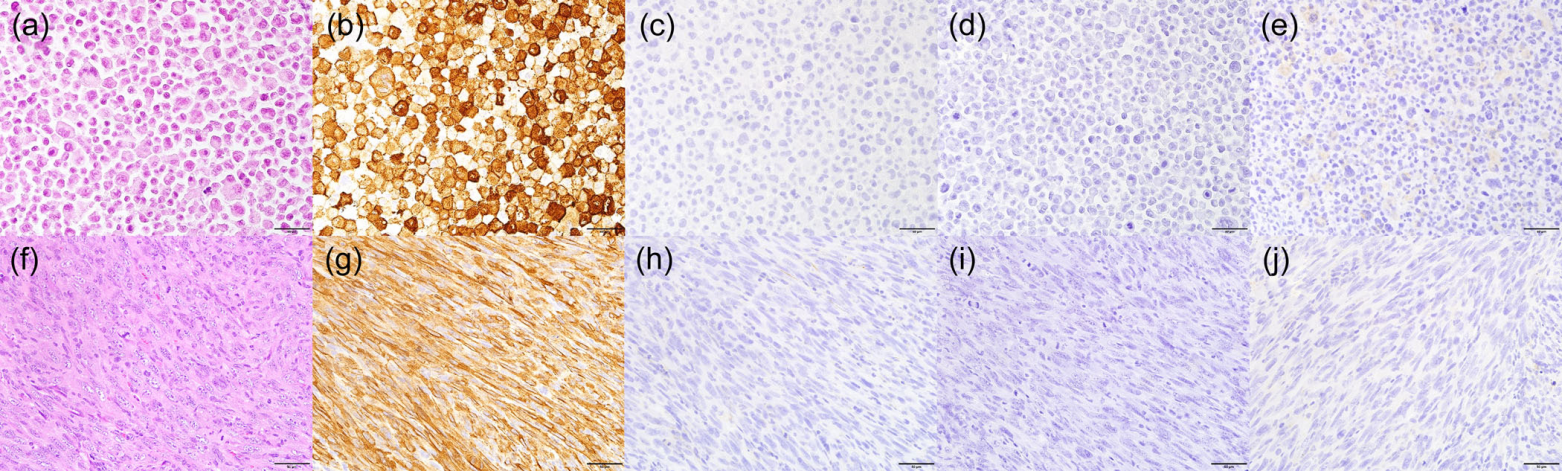
Histological findings of both the cell block of established cultured cells (a–e) and tumor formed after transplantation of the cells in mouse peritoneum (f–j). Tumor cells of the cell block and the transplanted tumor showed spindle‐shaped morphology, atypical nuclei, prominent nucleoli and abnormal mitosis in hematoxylin and eosin stained specimen (a, f). They were positive for α‐SMA (b, g), but negative for Desmin (c, h), CD34 (d, i), and KIT (e, j). Bars in Figure indicate 50 μm.

All mice transplanted with cultured cells in the peritoneal cavity had multiple foci of tumors on the peritoneum within two months (data not shown). Cell block of established cultured cells and the tumor formed after transplantation of the cells were histologically examined (Figure 4). Both of them showed that tumor cells were spindle‐shaped and had atypical and pleomorphic nuclei, prominent nucleoli, many abnormal mitoses and storiform growth pattern on the H&E staining (Figure 4a,f). IHC on both specimens revealed that the cells were positive for α‐SMA (Figure 4b,g), but negative for desmin (Figure 4c,h), CD34 (Figure 4d,i), KIT (Figure 4e,j) and DOG1 (data not shown). They were almost negative for S100 protein on the cell block specimen, but partially positive on the transplanted tumor (data not shown). Thus, the established cells were reminiscent of dedifferentiated GIST‐like cells and designated as DeGISTL1 cells.

### Effect of glutamine deprivation on proliferation of DeGISTL1 cells

**Figure 5:**
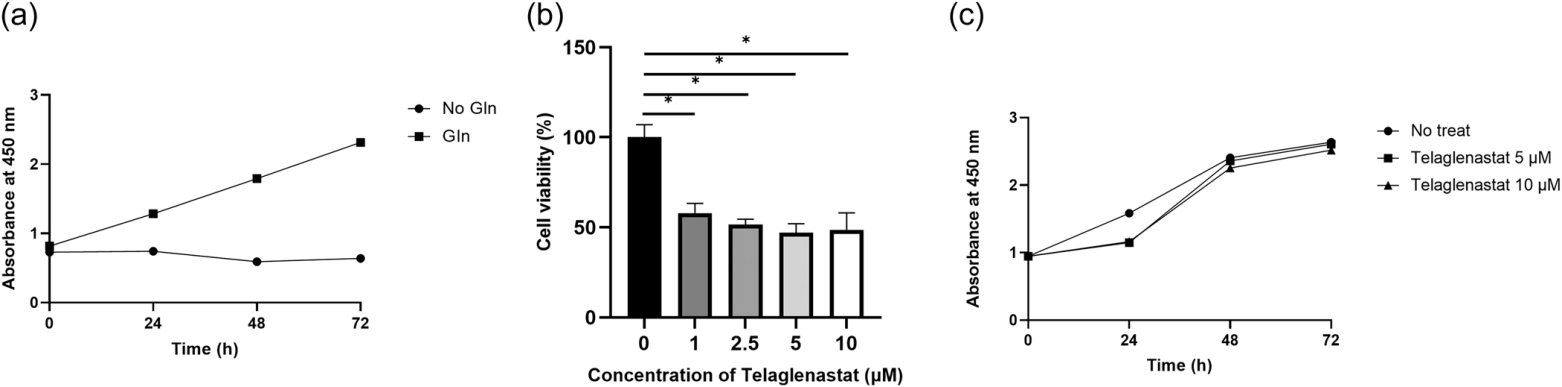
Effect of glutamine deprivation and Telaglenastat in DeGISTL1 cell proliferation. (a) Proliferation of DeGISTL1 cells was completely inhibited by glutamine deprivation. (b) Proliferation of DeGISTL1 cells was inhibited by Telaglenastat. (c) Proliferation of DeGISTL1 cells was inhibited by Telaglenastat at the concentration of 5 and 10 μM at 24 h after its addition. The effect was eventually lost after 24 h. Bars represent SDs of the means. *Means significant difference between groups at *p* < 0.05. ns, not significant.

To investigate the glutamine dependency of the DeGISTL1 cells showing high expression of metabolic pathway genes including *Gls*, effect of glutamine deprivation was examined. The proliferation of the DeGISTL1 cells was completely inhibited by glutamine deprivation, indicating that proliferation of the cells showed strong dependency on glutamine (Figure 5a).

### Effect of Telaglenastat on proliferation of DeGISTL1 cells

To confirm high glutamine dependency of the DeGISTL1 cells, effect of Telaglenastat (CB‐839), a GLS1 inhibitor, was examined. Proliferation of the DeGISTL1 cells (5 × 10^2^ cells/well) was inhibited dose‐dependently at various concentrations of Telaglenastat (1, 2.5, 5, and 10 μM) (Figure 5b) after 5 days of its addition. Proliferation of the DeGISTL1 cells (5 × 10^3^ cells/well) were inhibited by addition of 5 and 10 μM of Telaglenastat at 24 h although the effect was eventually lost (Figure 5c).

### Effect of Imatinib and Pimitespib on proliferation of DeGISTL1 cells

**Figure 6:**
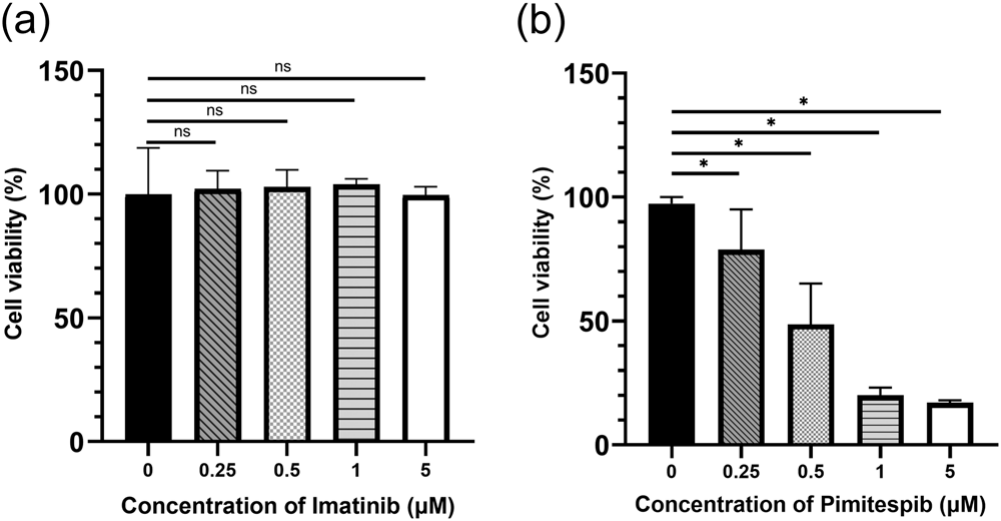
Effect of Imatinib and Pimitespib in DeGISTL1 cell proliferation. (a) Imatinib showed no effect on inhibition of DeGISTL1 cell proliferation at various concentrations (0.25, 0.5, 1, and 5 μM). (b) Pimitespib showed dose‐dependent inhibitory effect on proliferation of DeGISTL1 cells. Pimitespib almost completely inhibited DeGISTL1 cell proliferation at the concentration of 1 μM. Bars represent SDs of the means. *Means significant difference between groups at *p* < 0.05.

To examine the effect of TK inhibition on DeGISTL1 cells, Imatinib, one of TK inhibitors, was added to the culture media. In spite of relatively high expression of *PDGFRA* gene in DeGISTL1 cells, Imatinib showed no effect on inhibition of DeGISTL1 cell proliferation at various concentrations (0.25, 0.5, 1, and 5 μM) (Figure 6a). On the other hand, Pimitespib, one of HSP 90 inhibitors, showed dose‐dependent inhibitory effect on proliferation of DeGISTL1 cells showing high expression of HSP90 families (Figure 6b). Pimitespib almost completely inhibited DeGISTL1 cell proliferation at the concentration of 1 μM (Figure 6b).

### Effect of combination of Pimitespib and Telaglenastat on proliferation of DeGISTL1 cells

**Figure 7:**
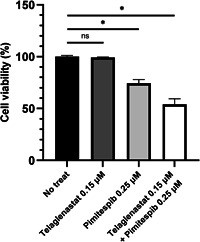
Effect of combination of Pimitespib and Telaglenastat in DeGISTL1 cell proliferation. Proliferation of the DeGISTL1 cells were additively inhibited. Bars represent SDs of the means. *Means significant difference between groups at *p* < 0.05.

Proliferation of DeGISTL1 cells was not apparently inhibited by 0.15 μM of Telaglenastat after 72 h culture, and significantly inhibited at the concentration of 0.25 μM of Pimitespib after 72 h culture. However, combination of 0.25 μM of Pimitespib and 0.15 μM of Telaglenastat showed synergistic inhibitory effect on proliferation of them after 72 h culture (Figure 7).

## DISCUSSION

We tried to establish a cell line which has the same characteristics of cecal GIST as that observed in KIT‐Asp818Tyr model mice, but both gene expression chip and IHC showed that the established cells abundantly expressed α‐SMA, and IHC revealed that they did not apparently express KIT and CD34, which are reliable markers of usual GIST. The phenotypes of the established cells without expression of any specific differentiation markers are considered to be dedifferentiated GIST‐like but not usual GIST‐like. They might be derived from dedifferentiation of cecal GIST cells,^4^ but there is a possibility that they are from non‐GIST cells within the cecal GIST tumor. Since dedifferentiated GISTs are usually extremely malignant, prognosis of the tumor is poor and drugs for its treatment are restricted.^4^ Thus, the new strategy for effective treatment is required for such highly malignant sarcomas.

The established DeGISTL1 cells showed prominent gene expression in metabolic pathway including GLS1 expression by KEGG pathway analysis, suggesting that their survival and growth is dependent on metabolic pathway. Although glutamine dependency of the cells is different in each cancer cell even in highly malignant cell, glutaminolysis is often important on survival and proliferation of highly malignant cancer cells.^8^ Therefore, we examined whether the glutamine metabolism is important for DeGISTL1 cells, which is likely to be highly malignant cancer cells with short doubling time. Deprivation of glutamine from the culture medium resulted in inhibiting the proliferation of them, confirming that glutamine metabolism is crucial for DeGISTL1 cell growth.

Telaglenastat is a GLS1 inhibitor with antitumor activity across various tumors, including renal cell carcinoma, triple‐negative breast cancer, non‐small cell lung cancer and lymphoma. However, effect of Telaglenastat in sarcomas remained to be clarified.^9^, ^10^, ^11^, ^12^ We examined the inhibitory effect of Telaglenastat on DeGISTL1 cell proliferation. We showed that proliferation of DeGISTL1 cells (5 × 10^2^) was dose‐dependently inhibited at 5 days and that proliferation of DeGISTL1 cells (5 × 10^3^) was inhibited at the concentration of 5 μM and 10 μM at 24 h after Telaglenastat addition. This result also suggested that DeGISTL1 cells were considered to be highly dependent on glutamine metabolism. The inhibitory effect of Telaglenastat on DeGISTL1 cells (5 × 10^3^) was not observed at 72 h after its addition probably because high dependency on glutamine metabolism of them might diminish its effect in a short time.

DeGISTL1 cells also showed high expression of HSP 90 families by gene expression chip. Their proliferation was considered to be supported by chaperone function of HSP90 families. Actually, Pimitespib, a HSP90 inhibitor which is approved in Japan for GISTs resistant to TK inhibitors including Imatinib, effectively inhibited proliferation of DeGISTL1 cells. On the other hand, Imatinib, an inhibitor of TK including KIT, did not show any effect on proliferation of them although they are derived from the cecal GIST of KIT‐Asp818Tyr model mice.

Since the single use of Telaglenastat or Pimitespib was effective for inhibition of DeGISTL1 cell proliferation, we examined whether combination of Telaglenastat and Pimitespib might show stronger effect. Proliferation of DeGISTL1 cells was additively inhibited by combination of them. HSP90 function might be closely related to glutamine metabolism. Anyway, these results suggested that proliferation of the human dedifferentiated GISTs might be effectively inhibited by Telaglenastat, Pimitespib, and combination of them.

Our study has some limitations. First, combination of Telaglenastat and Pimitespib showed additive effect on inhibition of DeGISTL1 cell proliferation, but the intimate mechanism could not be clarified. We have to examine the association between HSP90 function and glutamine metabolism. Second, only in vitro examination was carried out to show the effect of Telaglenastat, Pimitespib, and combination of them. We consider that in vivo experiments using mouse transplantation model are needed to show whether the administration of Telaglenastat, Pimitespib, and combination of them is realistic or not. Third, we did not clearly show the origin of DeGISTL1 cells. Since the cells have KIT‐Asp818Tyr mutation in genome level, they are apparently from the model mouse tissue. The cells might be derived from dedifferentiation of cecal GIST cells or from immature non‐GIST cells within the cecal GIST. Further examination is required to clarify their origin.

In conclusion, we established a dedifferentiated GIST‐like cell line, designated as DeGISTL1 cells, derived from cecal GIST in a mouse model of familial GISTs. This cell line might be a good model of dedifferentiated GIST cells, and useful to examine the effect of new drugs such as Pimitespib and Telaglenastat in dedifferentiated GIST patients. Combination of Pimitespib and Telaglenastat might become a possible effective candidate for treatment strategy in dedifferentiated GISTs. Since any cell lines with characteristics of usual GIST cells have not been established from cecal GISTs in familial GIST model mice yet, we have to continue to try to establish authentic GIST cell lines from them.

## AUTHOR CONTRIBUTIONS

Daisuke Sano, Takako Kihara, Yuan Jiayin, Neinei Kimura, Mizuka Ohkouchi, Yuka Hashikura and Shuichi Ohkubo carried out the experiments. Takako Kihara performed the genetical analysis. Daisuke Sano, Takako Kihara, and Seiichi Hirota evaluated biology of the cell line. Takako Kihara wrote the initial draft of the manuscript, and Seiichi Hirota supervised the writing of the manuscript. All authors have read and approved the final manuscript.

## CONFLICT OF INTEREST STATEMENT

Shuichi Ohkubo is a full‐time employee of Taiho Pharmaceutical Co., Ltd.

## Supporting information

Supplementary Figure 1 caption: Sequence data of the c‐*kit* exon 17 of the DeGISTL1 cells.
