# Supplementary figures and images for: Characterization of cell line with dedifferentiated GIST‐like features established from cecal GIST of familial GIST model mice
Source: Pathol Int. 2023 Feb 24;73(5):181–7. doi: 10.1111/pin.13315 (PMC11551817; doi:10.1111/pin.13315)

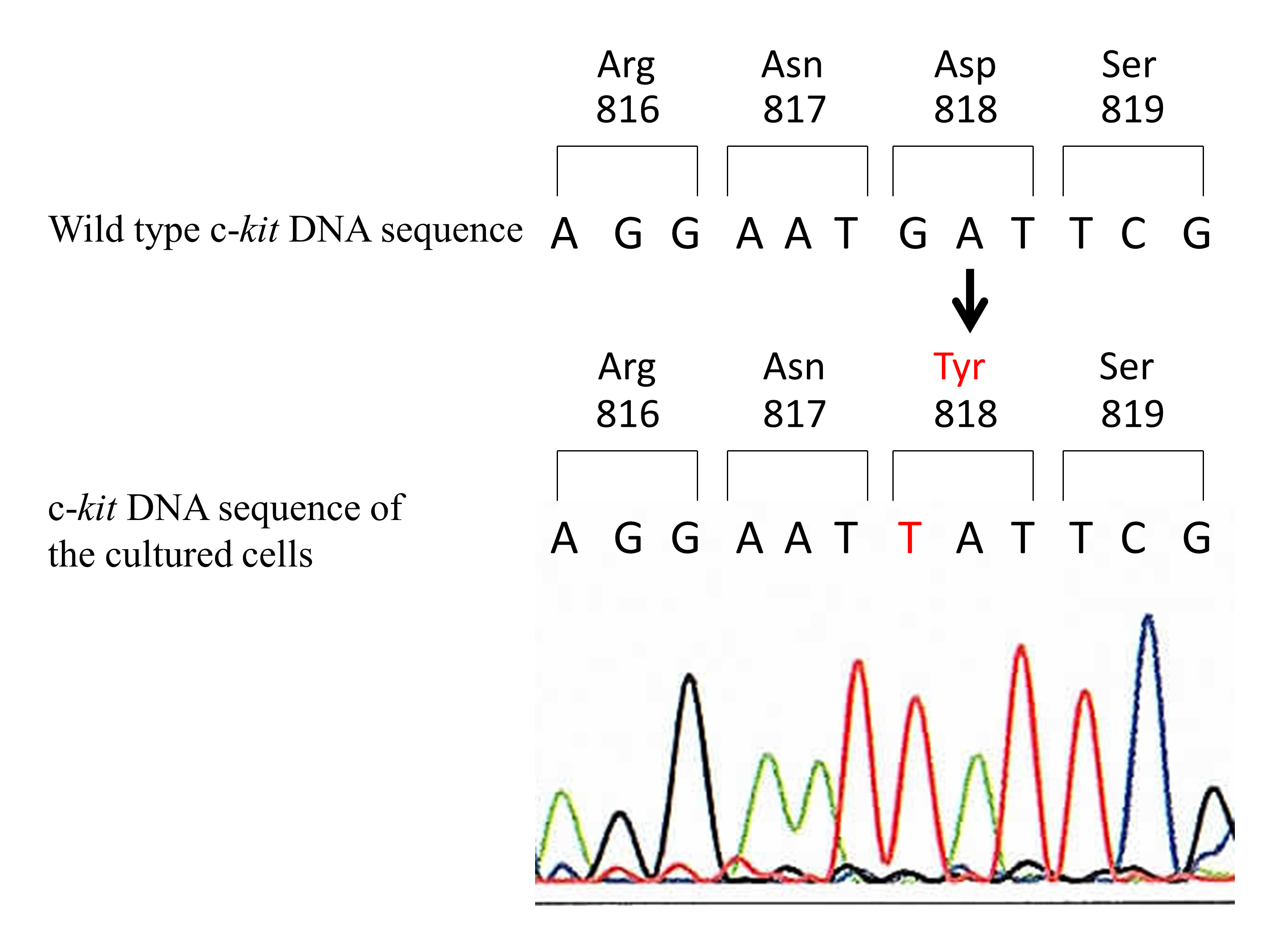

Supplement: Supplementary file 1 — Supplementary Figure 1 caption: Sequence data of the c‐kit exon 17 of the DeGISTL1 cells. [file PIN-73-181-s001.tif]
